# A Bayesian Perceptual Model Replicates the Cutaneous Rabbit and Other Tactile Spatiotemporal Illusions

**DOI:** 10.1371/journal.pone.0000333

**Published:** 2007-03-28

**Authors:** Daniel Goldreich

**Affiliations:** Department of Psychology, Neuroscience and Behaviour, McMaster University, Hamilton, Ontario, Canada; New York University, United States of America

## Abstract

**Background:**

When brief stimuli contact the skin in rapid succession at two or more locations, perception strikingly shrinks the intervening distance, and expands the elapsed time, between consecutive events. The origins of these perceptual space-time distortions are unknown.

**Methodology/Principal Findings:**

Here I show that these illusory effects, which I term perceptual *length contraction* and *time dilation*, are emergent properties of a Bayesian observer model that incorporates prior expectation for speed. Rapidly moving stimuli violate expectation, provoking perceptual length contraction and time dilation. The Bayesian observer replicates the cutaneous rabbit illusion, the tau effect, the kappa effect, and other spatiotemporal illusions. Additionally, it shows realistic tactile temporal order judgment and spatial attention effects.

**Conclusions/Significance:**

The remarkable explanatory power of this simple model supports the hypothesis, first proposed by Helmholtz, that the brain biases perception in favor of expectation. Specifically, the results suggest that the brain automatically incorporates prior expectation for speed in order to overcome spatial and temporal imprecision inherent in the sensorineural signal.

## Introduction

How does the brain interpret information from the senses? This unresolved question carries fundamental importance for neuroscience.

The brain faces a challenge as it attempts to translate sensory information into perception: Sensorineural activity imprecisely represents the physical world, In the case of tactile perception, spatial imprecision due to low receptor density poses a particular challenge, especially when brief stimuli preclude exploration. The most discriminating tactile sensors of primates, the fingertips, house a few hundred sensory nerve fibers per square cm [1], [2], a density four orders of magnitude lower than the peak ganglion cell density in the retina [3]. Without the benefit of exploratory movements, the fingertips' resolving power is on the order of one mm [4], [5], whereas the forearm has much worse acuity, resolving detail on the order of one cm [5]. Sensory systems face not only spatial, but also temporal imprecision, an expected consequence of stochastic variation in action potential timing, such as the several ms jitter in stimulus-evoked first-spike latencies of somatosensory cortical neurons [6].

A growing body of research suggests that the brain takes advantage of prior knowledge to enhance perceptual resolution beyond the limits imposed by sensorineural imprecision [7]. For example, the assumption that light originates from above disambiguates the retinal image, allowing the brain to more accurately perceive object shape from shading [8], [9]. Reliance on prior knowledge comes at a cost, however, as the rare physical event that violates expectation (e.g., a visual scene lit from below) is then misperceived. A percept that misrepresents physical reality–an illusion–is thus both a consequence of, and a clue to the brain's expectations regarding the world.

**Figure 1:**
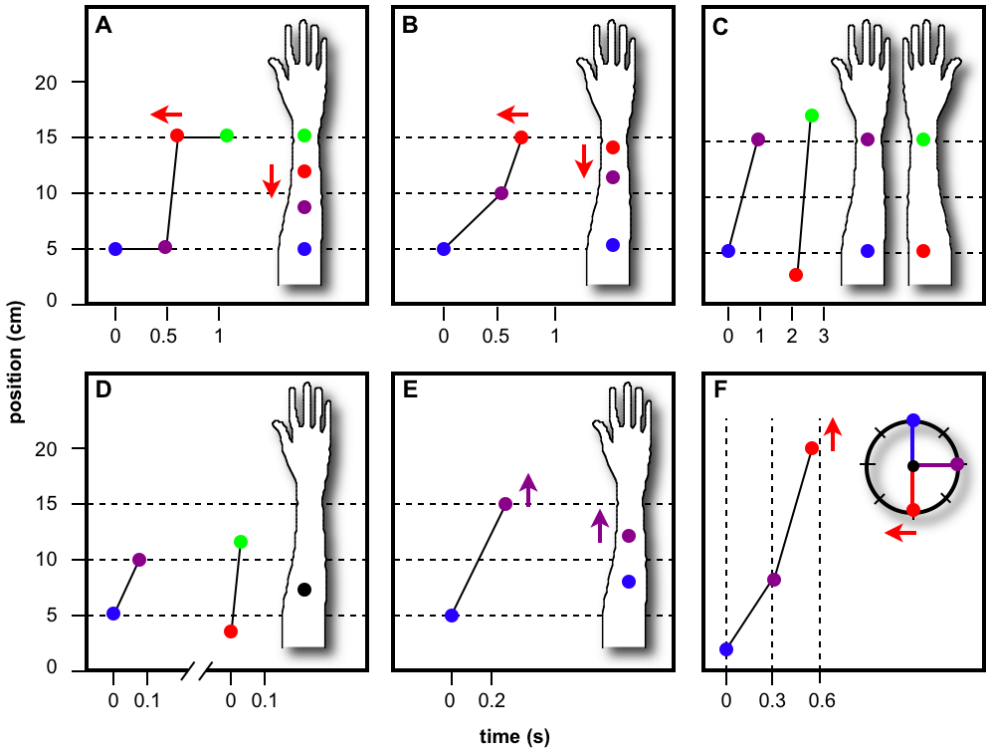
Tactile length contraction (A–E) and time dilation (F) illusions. Actual stimulus sequences (plotted points) evoke illusory perceived sequences (positions on forearms in A–E; clock times in F). Colored arrows in panels A, B, E, and F indicate direction of perceptual effect (arrow at right) caused by adjustment to corresponding stimulus location or time (arrow at left). (A) Rabbit illusion [12]. The two intermediate taps, separated by short temporal interval (rapid movement), are perceptually displaced towards one another. (B) Classic tau effect [15], [16]. The more rapidly traversed of two equal distances is perceived as shorter. (C) Tau effect with two-arm comparison [17]. Stimulus parameters were adjusted to reach the point of subjective equality, at which the greater distance (faster movement) is perceived equal to the shorter distance (slower movement). (D) Perceptual merging [18]. At very rapid velocities, the perceived locations of the two taps merge to a single point. The velocity required to accomplish perceptual merging increases with tap separation. (E) Two-stimulus distance estimation [19]. When inter-stimulus distance is increased at fixed inter-stimulus time, perceived distance both underestimates, and grows with, actual distance. (F) Kappa effect [21]. When inter-stimulus distance is increased at fixed inter-stimulus time, perceived inter-stimulus time overestimates actual time. Stimulus parameters were adjusted to reach the point of subjective equality, at which perception dilates the temporal interval defined by the greater distance (faster movement) to equal the slightly longer temporal interval defined by the smaller distance (slower movement).

Tactile perception is subject to characteristic spatiotemporal illusions. The best-known of these is the *cutaneous rabbit*, in which a sequence of three or more taps to two skin sites evokes the perception of an object hopping along the skin from the first site to the second, landing in the process on intervening skin that was never touched [10]–[14] (Fig. 1A). A vivid illusory tap occurs even when the intervening skin is anesthetized [11], revealing that the rabbit has its origins in the central nervous system, not in skin mechanics. Apparently related to the rabbit is the classic *tau effect*, in which the more rapidly traversed of two equal distances (defined by three stimuli) is perceived as shorter [15], [16] (Fig. 1B). Similarly, two different distances can be made perceptually equal simply by adjusting stimulus timing [17] (Fig. 1C). Even more remarkably, the perceived locations of two stimuli delivered in very rapid succession merge to a single point on the skin [18] (Fig. 1D). When stimulus timing is held constant, the perceived distance between stimuli both underestimates, and grows in proportional with, the actual distance [19], [20] (Fig. 1E). In the *kappa effect*, by contrast, the perceived time between stimuli dilates as the distance between stimuli is increased [21] (Fig 1F).

The above illusions apparently reflect just two fundamental perceptual distortions: underestimation of inter-stimulus distance (ISD), and overestimation of inter-stimulus time (IST). I term these distortions perceptual *length contraction* and *time dilation*, in analogy with the relativistic phenomena of those names [22]. Perceptual length contraction underlies many illusions [10]–[20], [23] (Fig. 1A–E). Perceptual time dilation, for reasons discussed below, has been less frequently reported [14], [21] (Fig. 1F). The present work proposes to explain the inferential process that generates these perceptual distortions. Related phenomena reported in vision [24] and audition [25] may share a similar explanation.

The Bayesian observer model described here replicates the spatiotemporal illusions illustrated in Figure 1. The model forms perceptual judgments by interpreting a spatially and temporally imprecise sensorineural signal in light of two plausible prior assumptions: 1) Stimuli separated by small spatial and temporal intervals originate from uniform object motion, and 2) objects that contact the skin tend to move slowly. As shown below, perceptual length contraction and time dilation are emergent properties of the Bayesian observer. When confronted with a fast stimulus sequence, the observer perceptually reduces ISD, and increases IST, reconciling velocity perception with expectation.

## Results

To infer which of many possible trajectories was taken by a sensed object, the Bayesian observer multiplies each candidate trajectory's *prior* (its probability, given only the expectation of slow movement) by its *likelihood* (probability of the sensorineural activity, given the trajectory) to obtain its *posterior* (probability of the trajectory, given sensorineural activity and expectation). The mode of the resulting posterior distribution, the most probable trajectory, is the percept: a compromise between imprecise sensorineural information and the observer's expectation of slow movement (see Materials and Methods for mathematical details).

### Basic Bayesian Observer

**Figure 2:**
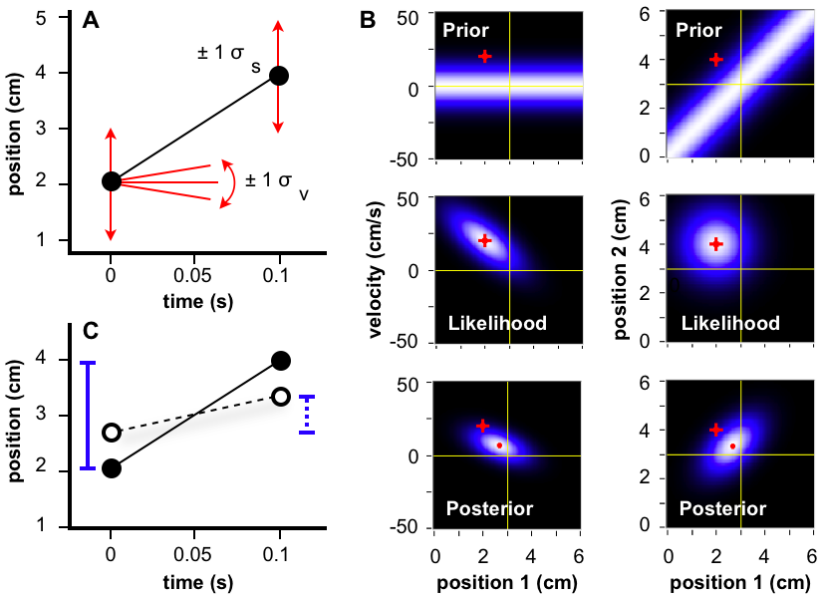
Basic Bayesian observer. (A) Two stimuli touch skin in rapid succession (filled circles). Reflecting sensorineural imprecision, each stimulus evokes a Gaussian likelihood function, centered on its actual position, with spatial standard deviation σ_s_ (vertical arrows: ±1 *σ_s_*). The observer considers slow movement most probable *a priori*, adopting a Gaussian prior probability distribution for velocity, centered on zero, with standard deviation *σ_v_* (slopes: ±1 *σ_v_*). (B) Candidate trajectories, represented by first stimulus position and velocity (left column) or, equivalently, first and second stimulus positions (right). Intensity represents probability. Prior (top) x likelihood (middle) ∝ posterior probability (bottom). The actual trajectory (red crosshairs in all panels) occupies the position of maximal likelihood, but its velocity exceeds prior expectation. Perception (mode of posterior; red dot) is a compromise between reality and expectation. (C) Actual (filled circles, solid line) and perceived (open circles, dashed line) trajectories. Perceived ISD (*l'* = 0.67 cm; dotted bar) underestimates actual ISD (*l* = 2 cm; solid bar), and perceived velocity (v' = 6.7 cm/s) underestimates actual velocity (v = 20 cm/s).

I first describe a basic version of the observer, which admits spatial but not temporal imprecision (Fig. 2). This model experiences length contraction but not time dilation. The observer's perceived ISD, *l'*, is related to actual ISD, *l*, and IST, *t*, by the length contraction equation (for derivation, see Materials and Methods):
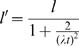
1where *λ* = *σ_v_*/*σ_s_* is the single free parameter of the model (see Fig. 2A).

**Figure 3:**
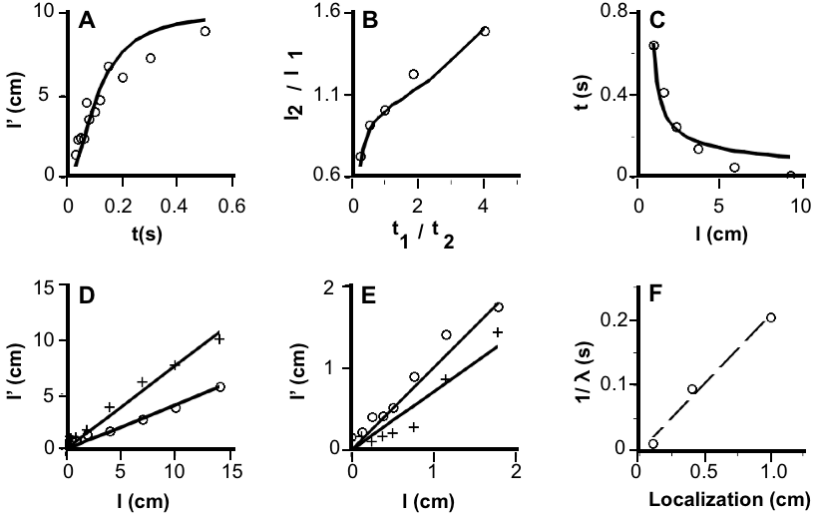
Human data from five studies (symbols) and basic Bayesian observer's performance on the same tasks (solid curves in A–E). For each study, the value of λ was chosen to minimize the mean-squared error between model and human performance. (A) Rabbit on forearm (Fig. 1A) [12]. R^2^: 0.80. λ: 12.7/s. (B) Two-arm tau effect (Fig. 1C) [17]. x-axis: IST ratio ( pair 1/pair 2 ). Pair 1 ISTs (from left to right) were 0.2, 0.35, 0.5, 0.65, and 0.8 s; pair 2 IST = 1.0 s-pair 1 IST. y-axis: ISD ratio ( pair 2/pair 1 ) that resulted in equality of perceived ISDs ( pair 1 *l'* = pair 2 *l'* ). Pair 1 ISD was fixed at 10 cm. R^2^: 0.95. λ: 9.4/s. (C) Perceptual merging experiment (Fig. 1D) [18]. R^2^: 0.92. *λ*: 4.2/s. (D) Two-stimulus distance estimation for longitudinally separated stimuli on forearm (circles) and horizontally separated stimuli on forehead (crosses) at 0.24 s IST (Fig. 1E) [19]. Forearm R^2^: 0.94. Forehead R^2^: 0.90. Forearm *λ*: 4.9/s. Forehead *λ*: 10.5/s. (E) Two-stimulus distance estimation for longitudinally separated taps to the index finger [20]. Circles: 1.1 s IST; crosses: 26 ms IST. R^2^ (1.1 s): 0.94, R^2^ (26 ms): 0.90. *λ*: 85.1/s. (F) Point localization accuracies for finger, forehead, and forearm [5] plotted against 1/*λ* (dashed line). R^2^: 0.99.

Equation 1 predicts that perceived ISD will: 1) underestimate actual ISD; 2) asymptotically approach actual ISD as IST increases; and 3) increase linearly with actual ISD, at constant IST. Each of these predictions is borne out by the human perceptual data. Indeed, the Basic observer model explains between 80 and 95% of the variance in the data from five studies of tactile length contraction illusions (Fig. 3A–E).

Figure 3A shows rabbit illusion data [12] (Fig. 1A). As predicted by Equation 1, perceived ISD between the second and third taps to the forearm asymptotically approached actual ISD (10 cm) as IST was increased.

Figure 3B shows two-arm tau effect data [17] (Fig. 1C). For each pair-1 to pair-2 IST ratio, the pair-2 ISD was found that was perceptually equal to the fixed, 10-cm pair-1 ISD. In agreement with Equation 1, relatively shorter pair-2 ISTs (t_1_/t_2_>1) required relatively larger pair-2 ISDs (l_2_/l_1_>1) as the condition for perceptual equality.

Figure 3C shows perceptual merging data [18] (Fig. 1D). At each ISD, the IST was determined for which two electrocutaneous pulses to the forearm became spatially indistinguishable. For modeling purposes, the assumption was made that this occurs when perceived ISD drops below a threshold value. The data were best fit with a perceived ISD threshold of 0.8 cm, a sensible value given that the point localization accuracy of the human forearm is approximately 1 cm [5]. As predicted by Equation 1, larger ISDs required shorter ISTs.

Figure 3D shows perceived distance between two electrocutaneous pulses at fixed IST [19] (Fig. 1E). As predicted by Equation 1, perceived and actual ISD correlated linearly. Note also that the forehead showed less perceptual length contraction than did the forearm (see Lambda Variation below).

Figure 3E shows perceived distance between two taps to the index finger, determined at two ISTs [20]. As predicted by Equation 1, less length contraction occurred at the longer IST, and perceived and real ISD correlated approximately linearly. The data at the shorter IST suggest a slight nonlinearity, a result predicted by the full Bayesian observer model (below).

### Lambda Variation

A small *λ* results from strong expectation for slow movement (small *σ_v_*) and/or poor spatial acuity (large *σ_s_*), either of which facilitates perceptual length contraction (Equation 1). Conversely, when *λ* is large, less length contraction occurs. The model's replication of human data shows that the value of *λ* varies from one body region to another. Length contraction is most pronounced on the forearm (Figs. 3A–D, average *λ*: 7.8/s), somewhat less pronounced on the forehead (Fig. 3D, *λ*: 10.5/s), and least pronounced on the finger (Fig. 3E *λ*: 85.1/s). Is this variation in *λ* due to variation in *σ_s_,* in *σ_v_*, or both?

The value of *σ_s_* is reflected in the accuracy with which humans localize a single point stimulus, an indicator of tactile acuity that has been mapped throughout the body surface [5]. Therefore, a linear relation between point localization accuracy and *1/λ* would suggest that *σ_v_* remains constant throughout the body surface, and that *λ* variation is caused by variation in *σ_s_*; conversely, a nonlinear relationship would indicate variation in *σ_v_*.

Figure 3F applies this reasoning to the two studies that reported perceived vs. real distance (Figs. 3D, E). Since these used similar perceptual tasks, differences in *λ* are attributable primarily to body region. The model's best-fit *λ* values for these studies are 4.9, 10.5, and 85.1/s, for forearm, forehead, and finger, respectively. The corresponding point localization accuracies, approximately 1, 0.4, and 0.1 cm [5], indeed correlate linearly with *1/λ* (Fig. 3F), strongly suggesting that the low-velocity prior, *σ_v_*, is conserved from one body region to another, and that variation in *λ* with body region results from variation in tactile acuity (*σ_s_*) alone.

### Temporal Order Judgment

The mode of the posterior probability distribution is the trajectory that the model “perceives;” however, the mode represents only a single point from the full posterior distribution (Fig. 2B). If the brain, like the model, could access the full distribution, what sort of additional information would be in its possession?

Access to the full posterior distribution would allow the formulation of probabilistic perceptual inferences, such as the perceived probability that movement occurred in one or the other direction. This probability is not available from the mode of the posterior distribution, but is readily obtained from the full posterior distribution by integration.

**Figure 4:**
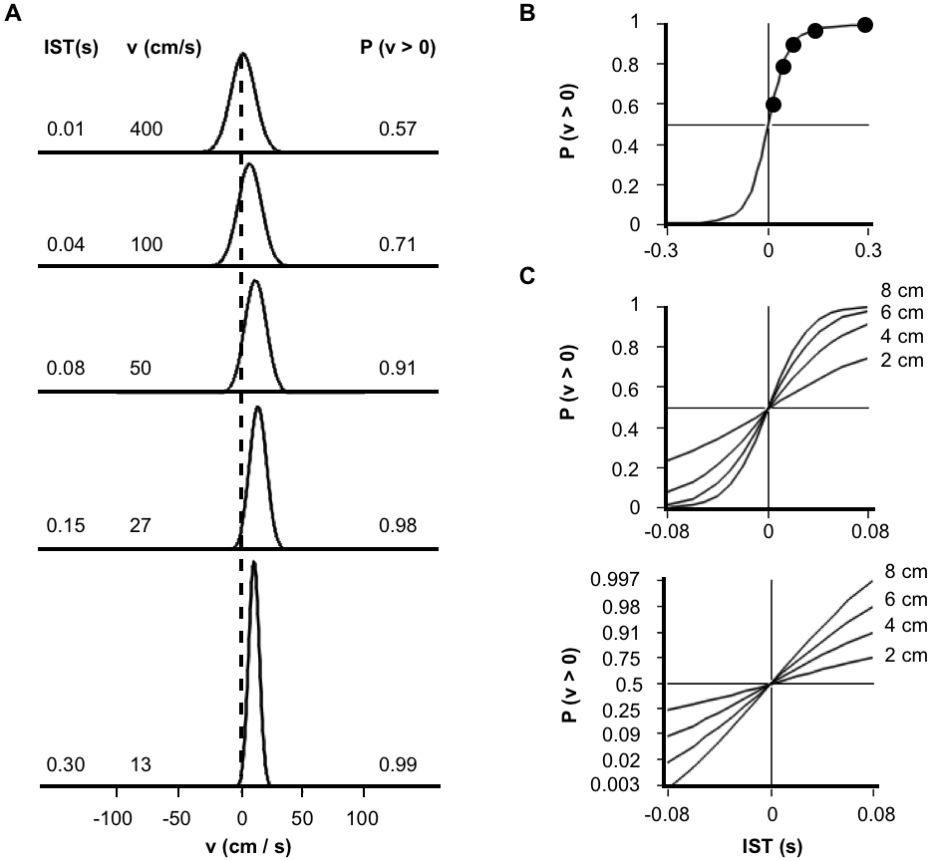
Temporal order judgments of the basic Bayesian observer. (A) Posterior probability distributions for velocity, for 4 cm ISD and 0.01 s-0.30 s ISTs, obtained by integrating across the corresponding 2-dimensional posterior probability distributions (e.g., Fig. 2B, lower left). A second integration finds the area under each curve to the right of zero, *P(v>0)*. (B) TOJ curve, plotting *P(v>0)* from (A), and additional values for the opposite movement direction (negative x-axis), against IST. (C) Upper panel: TOJ curves for 2 cm to 8 cm ISD, and −80 ms to 80 ms IST. Lower panel: The same curves plotted with y-axis probit (cumulative normal probability) coordinate spacing. As with human TOJ curves plotted in this manner [26]–[28], these curves are linear. Model parameter values used for all panels: *σ_s_*, 1 cm; *σ_v_* , 10 cm/s.

This integration can be viewed as a two-step process. First, the posterior probability distribution (Fig. 2B, lower left) is integrated at each value of velocity (y-axis) across all values of first stimulus position (x-axis). This yields a posterior probability distribution for velocity (Fig. 4A). Next, the velocity distribution is integrated to the right of zero, yielding the perceived probability that the velocity was positive, *P(v>0)*.

Since positive velocity indicates movement in a particular direction, for instance distally along the forearm (see Fig. 1), *P(v>0)* represents a graded opinion regarding the direction of motion, or equivalently, a graded answer to the question: “Which stimulus (distal or proximal) came first?” Interestingly, *P(v>0)*, plotted against IST, (Fig. 4B) resembles a human temporal order judgment (TOJ) curve, which plots against IST the percent of correct responses to this same question [26]–[28].

It may seem surprising that the basic observer model, which accurately registers the time of occurrence of each stimulus, nevertheless remains uncertain as to stimulus order (0<*P(v>0)<1*). This situation arises because, although the model knows *when* each stimulus occurred, it is uncertain *where* the stimulus occurred (see Fig. 2B, lower right), and consequently it is uncertain about which location (e.g., distal or proximal) was stimulated first.

Interestingly, for a given IST, the model grows more confident of stimulus order as ISD increases; equivalently, the model's TOJ *threshold* [27] or *just-noticeable difference* [28], the IST at which *P(v>0) = 0.75*, decreases with increasing ISD (Fig. 4C). Intriguingly, this influence of ISD agrees qualitatively with results from several human perceptual studies [27]–[29]. For instance, TOJ thresholds on the thigh decrease by several ms when ISD is doubled from 10 to 20 cm [27].

Also in agreement with human data [26]–[28], the model's TOJ curves (for–0.08 s to 0.08 s IST) are linear when transformed to probit (cumulative normal) coordinates (Fig. 4C, lower). This linearity arises because the model's posterior probability distribution for velocity maintains a nearly fixed Gaussian shape as it shifts nearly linearly to the right with increasing IST (Fig. 4A, upper three plots).

These points of concordance between human and model TOJ performance suggest that the brain indeed integrates across the full posterior probability distribution. However, more detailed human data are needed to quantitatively compare to the model's TOJ performance.

### Spatial Attention

Figure 2C shows that the basic Bayesian observer perceives the first and second stimulus positions as shifted by equal distances in opposite directions, such that the perceived and actual trajectories share the same midpoint. In one circumstance, however, this prediction does not match human perception: When instructed to focus their attention on one of the two stimulus locations, humans report a smaller perceptual shift for taps at that location than at the other. The midpoint of the perceived trajectory thus shifts towards the attended location [12].

**Figure 5:**
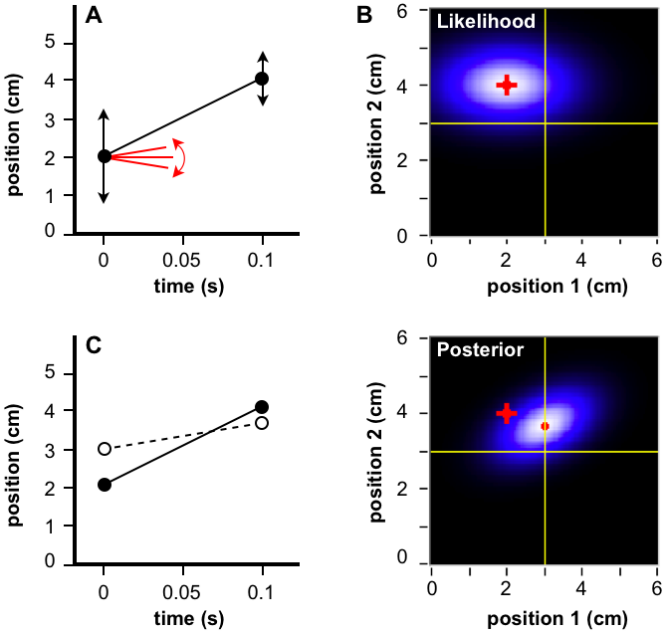
Basic Bayesian observer with directed spatial attention. (A) Plot of the same stimuli (filled circles) shown in Figure 2. Attention directed to the location of the second stimulus lowers *σ_s2_* and increases *σ_s1_* (vertical arrows: ±1 *σ_s_*). The observer considers slow trajectories most probable *a priori* (red slopes: ±1 *σ_v_*). (B) Likelihood and posterior distributions in positional trajectory space (The prior is identical to that shown in Fig. 2). The oval-shaped likelihood distribution results because *σ_s1_*≠*σ_s2_*. The mode of the posterior (red dot) shows that the perceived location of the first stimulus has shifted more than that of the second stimulus, relative to their actual locations (red crosshairs). (C) Actual (filled circles, solid line) and perceived (open circles, dashed line) trajectories. The midpoint of the perceived trajectory has shifted towards the location of stimulus 2 by 0.3 cm relative to the actual trajectory midpoint. Model parameter values used for all panels: *σ_s1_*, 1.23 cm; *σ_s2_*, 0.70 cm; *σ_v_* , 10 cm/s.

This result is reproduced by the basic Bayesian observer if attention directed towards one location reduces spatial uncertainty there (Fig. 5). The modulation of somatosensory cortical neuronal activity by spatial attention [30]–[32] provides a plausible mechanism for this local refinement of tactile acuity. The influence of spatial attention on the Bayesian observer is graded. The greater the attentional imbalance between the two locations, the more closely the perceived trajectory midpoint approaches the preferentially attended location (see Materials and Methods).

### Full Bayesian Observer

The basic observer accurately registers the time of occurrence of each stimulus, and therefore perceives IST veridically. However, some studies indicate that perceived IST increases subtly as ISD is lengthened. For instance, in a point-of-subjective-equality experiment [21], two taps to the forearm at 12 cm ISD, 269 ms IST evoked the same perceived IST as taps at 6 cm ISD, 308 ms IST (Fig. 1F). This time dilation illusion, the *kappa effect* [14], [21], has been studied much less extensively than the length contraction illusions considered above, and is reportedly less robust [11].

**Figure 6:**
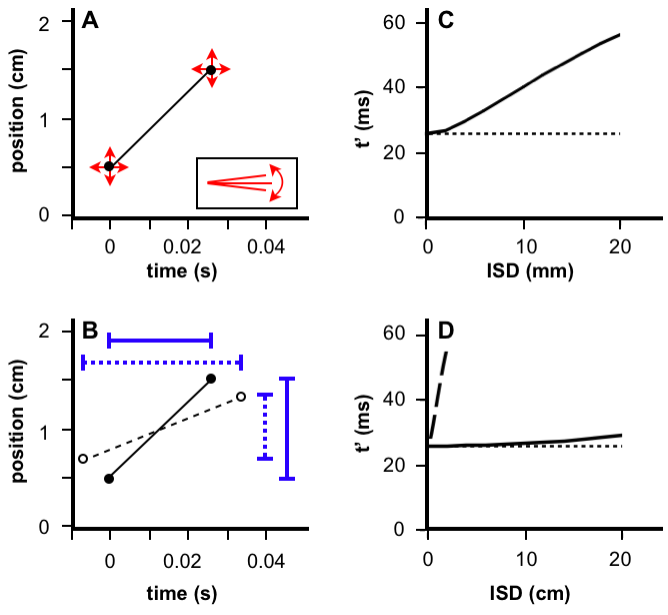
Full Bayesian observer. (A) Two stimuli (filled circles) touch the fingertip in rapid succession. The observer is uncertain as to stimulus location (vertical arrows: ±2 *σ_s_* for clarity) and time of occurrence (horizontal arrows: ±1 *σ_t_* ), and considers slow movement most probable *a priori* (inset slopes: ±1 *σ_v_*). (B) Actual (filled circles, solid line) and perceived (open circles, dashed line) trajectories. Perception underestimates ISD (*l'* = 0.64 cm <*l* = 1 cm; vertical bars) and overestimates IST (*t'* = 40 ms>*t* = 26 ms; horizontal bars). (C) Perceived IST on finger dilates as ISD increases from 0–20 mm (solid line; kappa effect). The basic observer, by contrast, perceives IST veridically (dotted line). (D) Time dilation of full observer on forearm for 0–20 cm ISD (solid line). Perception on finger (C) is reproduced for comparison (dashed line). All panels: IST, 26 ms; *σ_t_,* 5 ms; *σ_s_* (finger), 1 mm; *σ_s_* (forearm), 1 cm; *σ_v_*, 4.7 cm/s.

The kappa effect is reproduced by the full Bayesian observer model, in which tactile sensation suffers from temporal as well as spatial uncertainty (Fig. 6A). The full observer experiences perceptual time dilation as well as length contraction (Fig. 6B). Furthermore, it experiences increasing time dilation as ISD increases at fixed IST (Fig. 6C), the hallmark of the kappa effect.

What causes the kappa effect? As ISD is lengthened, the trajectory velocity (slope in Fig. 6A) increases. Like the basic observer, the full observer is inclined by its slow-movement expectation to perceptually reduce trajectory slope. However, the full observer has not one but two ways to accomplish this. The steeper a line segment, the more efficiently its slope is reduced by horizontal expansion (time dilation) compared to vertical compression (length contraction). An emergent property of the model, then, is that it relies more heavily on time dilation as ISD increases.

Why has the kappa effect, a time dilation illusion, been more elusive than the rabbit, the tau effect, and other length contraction illusions? The Bayesian observer provides a simple explanation: Most studies of tactile spatiotemporal illusions, and all studies of the kappa effect, have utilized the forearm. Due to its poor spatial resolution, the forearm is an ideal choice for investigations of length contraction illusions, but, for the same reason, the model experiences a very small kappa effect on the forearm (Fig. 6D). Where tactile spatial acuity is poor (e.g. forearm; large *σ_s_*), length contraction readily reconciles perception with prior expectation. Only where spatial acuity is relatively good (e.g. fingertip; small *σ_s_*) does time dilation necessarily play a greater role.

The length contraction equation for the full observer is: 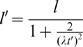
2


**Figure 7:**
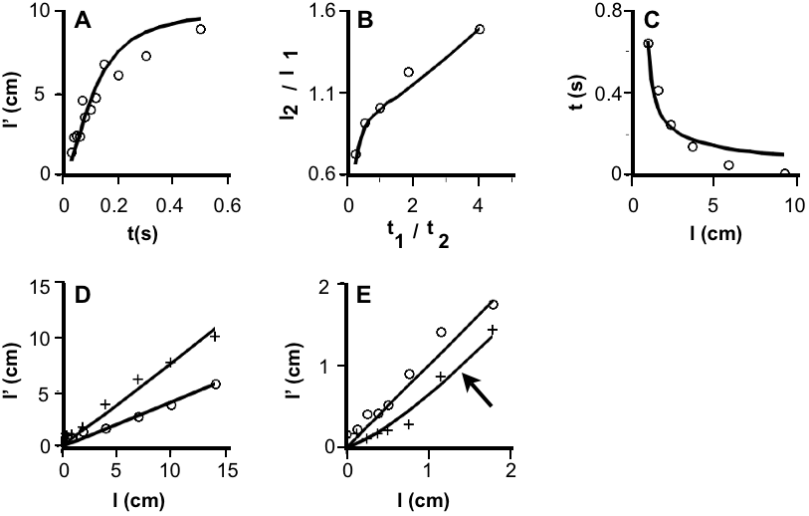
Human data from five studies and full Bayesian observer's performance on the same tasks. The same five data plots shown in Fig. 3 (symbols) are reproduced here along with performance of the full model (curves). *σ_t_* was fixed at 5 ms, *σ_s_* set to 1 cm (forearm) or 0.1 cm (finger), and the value of λ adjusted in each case to minimize the mean-squared error between model and human performance. The performance of the full model is very similar to that of the basic model (compare to Fig. 3A–E). However, perception on the finger at 26 ms IST (crosses in E) is better-matched by the nonlinear performance of the full model (arrow; R^2^: 0.95) than by the linear performance of the basic model (R^2^: 0.90; compare Fig. 3E).

**Figure 8:**
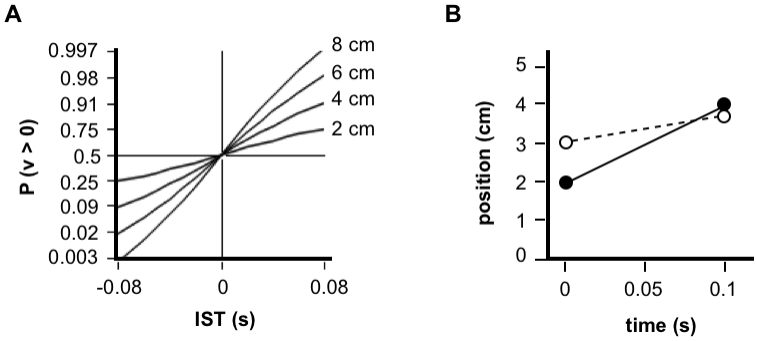
Temporal order judgment and spatial attention effects of the full Bayesian observer. (A) TOJ curves for 2 cm to 8 cm ISD, and −80 ms to 80 ms IST, plotted with y-axis probit coordinate spacing (compare to Fig. 4C lower). Model parameter values used: *σ_s_*, 1 cm; *σ_v_*, 10 cm/s; *σ_t_*, 5 ms. (B) Actual (filled circles, solid line) and perceived (open circles, dashed line) trajectories when the full observer directs attention to the location of the second stimulus (compare to Fig. 5C). Model parameter values used: *σ_s1_*, 1.23 cm; *σ_s2_*, 0.70 cm; *σ_v_*, 10 cm/s; *σ_t_*, 5 ms.

Equation 2 resembles Equation 1, but substitutes perceived IST, *t'*, for actual IST, *t.* Because *t'* increases with *l* (the kappa effect), Equation 2, unlike Equation 1, predicts a nonlinear relationship between perceived and real ISD. This nonlinearity will be most pronounced (but still subtle) when the kappa effect is at its strongest; that is, for fast trajectories on body areas with fine tactile acuity. This prediction is consistent with the subtly nonlinear relationship observed between perceived and real ISD on the fingertip, at 26 ms IST (Fig. 3E, crosses). The full model fits these data (Fig. 7E, crosses) better than does the basic model, while its perception of slower trajectories on the fingertip (Fig. 7E, circles) and its perception on body areas other than the fingertip (Fig. 7A–D, Fig. 8), is nearly indistinguishable from that of the basic model.

### Perceived Velocity

The perceived velocity evoked by two punctate tactile stimuli has yet to be measured experimentally. The basic Bayesian observer's perceived velocity, *v'* = *l'/t*, is given by (see Materials and Methods):
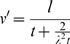
3


**Figure 9:**
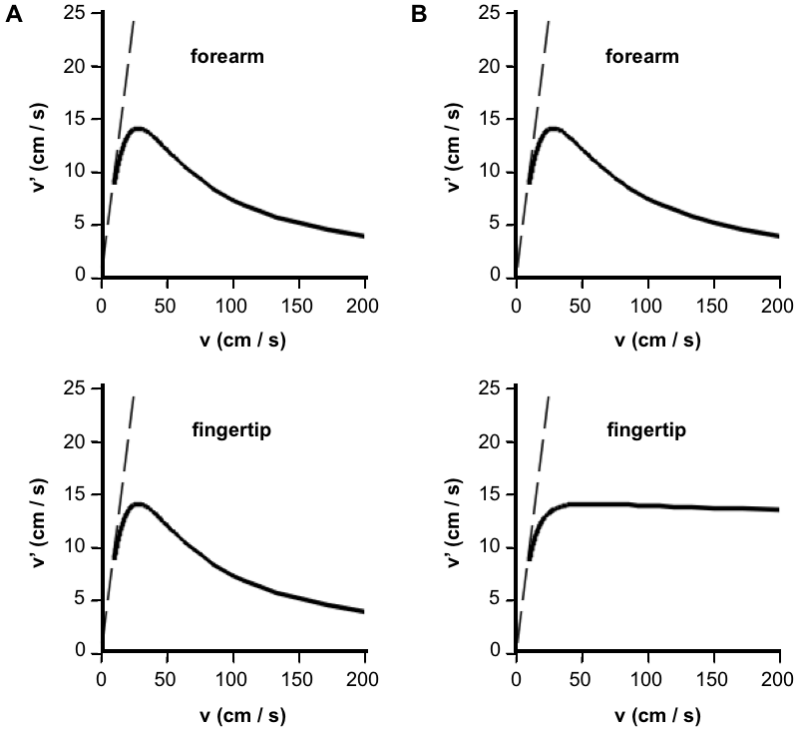
Velocity perception of the Bayesian observer models. Perceived velocity, *v'*, is plotted against real velocity, *v*, for the basic (A) and full (B) Bayesian observer models, on both forearm (top panels) and fingertip (bottom panels). In all cases, real velocity was increased by reducing IST at fixed ISD (4 cm for forearm; 4 mm for fingertip). (A) Basic observer: Perceived velocity, *v'* = *l'*/*t*, was derived from Equation 3. Real velocity v* = 28.28 cm/s (Equation 4) results in peak perceived velocity v'_max_ = 14.14 cm/s (Equation 5). (B). Full observer: Perceived velocity, *v'* = *l'*/*t'*, was determined from Equations 2 and 16, with *σ_t_* set to 5 ms. Dotted lines in all panels: x = y. (A) and (B): *σ_s_* was set to 1 cm (forearm) or 1 mm (finger), and *σ_v_* to 10 cm/s.

This equation shows that perceived velocity underestimates real velocity, *v* = *l/t*. Interestingly, the equation also predicts that real and perceived velocities will relate non-monotonically when IST is reduced at fixed ISD (Fig. 9A). Thus, the Bayesian observer experiences a perceptual speed limit. Perceived velocity, *l'*/*t*, initially grows as IST, *t*, decreases. However, as IST is progressively reduced, the retarding effect of the consequent length contraction (reduction in *l'*; Equation 1) counters and eventually overcomes the effect of IST reduction, so that perceived velocity diminishes. Indeed, perceived velocity peaks at real velocity, v*, given by
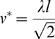
4and the maximum perceived velocity, v'_max_, equals half v*:
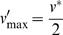
5The full Bayesian observer's perceived velocity, v' = l'/t', peaks similarly, but falls off more slowly than does the basic observer's perceived velocity (Fig. 9B). Once again, this difference between the two models is most pronounced where tactile acuity is greatest (e.g., the fingertip).

## Discussion

Tactile spatiotemporal illusions have long intrigued and puzzled researchers. Perhaps the earliest description was made by Weber, who in 1834 reported that the perceived separation between two fixed caliper points expands as the points are dragged along the skin from the forearm towards the fingertips [33]. Weber concluded, in agreement with modern studies [19], [20], [34], that distance is underestimated on skin regions with poor tactile acuity, a phenomenon termed *spatial compression* by Green [34]. Some 100 years after Weber's publication, Helson [15] described the tau effect, showing that perceived tactile distance depends on inter-stimulus timing. The rabbit illusion later described by Geldard and Sherrick [10] confirmed the temporal dependence of spatial perception, while the kappa effect, described concurrently by Cohen and colleagues [24] in vision and Suto [21] in touch, revealed the spatial dependence of temporal perception.

Several clever theoretical explanations have been advanced to account for these illusions. Collyer [35], [36] proposed that the brain expects movement to occur at the same velocity in all segments of a multi-segment stimulus sequence, and that it adjusts space and time perception accordingly. For instance, the classic tau effect (Fig. 1B) was hypothesized to arise because the brain expects movement to occur at the same velocity between the first and second, as between the second and third stimulus positions. A related line of reasoning was followed by Jones and Huang [37], who modeled perceived inter-stimulus distance and time as weighted averages of actual and expected inter-stimulus distance and time, with the expected values derived from a constant velocity assumption. A different and particularly creative approach was taken by Brigner, who hypothesized that spatiotemporal illusions result from rotation of a perceptual space-time coordinate frame [38], [39]. The hypothesized transformation achieves spatial and temporal perceptual adjustments in a way that is, roughly, the converse of that shown in Figure 6B: The trajectory line (filled circles) remains fixed, while the space and time axes rotate together counterclockwise.

None of these interesting explanations has been applied quantitatively to a wide variety of experimental data, and each has shortcomings. Collyer's hypothesis may prove relevant to the perception of sequences with three or more stimulus locations, but its application to sequences with just two spatial positions, which also produce illusions (e.g., Fig. 1A), is less clear. The weighted average model proposed by Jones and Wang leaves unanswered the question of how the relative weights are determined, and particularly what mechanism governs their evident dependence on the duration of the stimulus sequence. Brigner's intriguing proposal is able to explain, at least qualitatively, perceptual illusions evoked by stimuli at just two positions, but how or why the brain would undertake the proposed coordinate transformation is unclear.

The Bayesian observer model described here provides a coherent explanation for perceptual length contraction and time dilation, and replicates the rabbit illusion, the tau effect, the kappa effect, and a variety of other spatiotemporal illusions. The results suggest that the brain takes advantage of the expectation for slow speed, presumably based in tactile experience, to improve perception beyond the limits imposed by spatial and temporal uncertainty inherent in the sensorineural signal.

The Bayesian observer's slow-speed expectation recalls a visual model with that expectation that reproduces contrast effects on motion perception [40]. The remarkable explanatory power of these models supports Helmholtz's view of perception as a process of unconscious inference, in which “previous experiences act in conjunction with present sensations to produce a perceptual image” [41]. The perceptual space-time distortions that emerge from the Bayesian observer, and characterize human tactile perception, are loosely analogous to the physical length contraction and time dilation described in the Special Theory of Relativity [22]. I do not attach special significance to this analogy, but note simply that it arises because any postulated constraint on speed naturally yields distortions of space and/or time.

The Bayesian observer makes several novel testable predictions and suggests many experiments. For example, the model predicts more pronounced time dilation (Fig. 6), as well as less pronounced length contraction (Fig. 3), on body areas with finer tactile acuity, and it predicts a perceptual speed limit on the velocity evoked by dual punctate stimuli with fixed spacing (Fig. 9). Temporal perception experiments will determine whether the kappa effect is indeed more pronounced on body areas with finer tactile acuity (Fig. 6D), while velocity perception experiments will provide data for comparison to the curves shown in Figure 9B. In addition, the model suggests experiments with within-subjects designs to determine the contributions of σ_s_ and σ_v_ to variation in λ, not only across body regions (Fig. 3F), but also across perceptual tasks and as a result of perceptual learning. Finally, although designed to model tactile perception, the Bayesian observer may prove relevant to perception in other sensory modalities that show similar spatiotemporal illusions. For instance, Figure 6D, translated to visual perception, predicts a greater kappa effect for foveal than peripheral stimulus sequences.

Important work related to the model remains to be done. Experiments are needed to determine the precise shapes of the prior and likelihood distributions assumed by human observers as they perceive tactile stimulus sequences, as has been done for visual motion perception [42]. The Gaussian priors and likelihoods used in the model may need to be refined as a result of such experiments. Furthermore, theoretical work is needed to extend the model to treat the perception of more complex punctate stimulus sequences (e.g., [43], [44]), and of smoothly moving objects [45], [46]. Interestingly, humans progressively underestimate the fixed distance traversed by a brush swept briskly across the skin as sweep duration decreases [45], a result in qualitative agreement with Equation 1.

Finally, research is needed to determine where in the brain the Bayesian probability distributions hypothesized to serve tactile perception are represented, and by what neural mechanism they are generated. Interestingly, topographically appropriate somatosensory cortical activity accompanies illusory rabbit percepts on the forearm [47]. Research is needed, then, to explore connections between models of somatosensory cortical function recently proposed to account for the rabbit illusion [14], [48], and hypothesized neural representations of Bayesian probability distributions [49].

## Materials and Methods

### Basic Model (Fig. 2)

Each candidate trajectory was described by a velocity (slope) *m*, and first stimulus position (y-intercept), *b*.

Bayes' theorem relates the posterior probability of the candidate trajectory, given stimulus-evoked neural data, *D*, *P(m,b*|*D)*, to the trajectory's prior probability, *P(m,b)*, and likelihood, the probability of the stimulus-evoked neural data given the trajectory, *P(D*|*m,b)*:
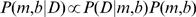
6


The prior, *P(m,b)*, was represented by a Gaussian distribution for trajectory velocity, centered at zero, to reflect the observer's expectation of slow movement. *P(m,b)* was independent of *b*, because a uniform prior (no constraint) was assumed for *b*:
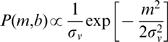
7


The likelihood, *P(D*|*m,b)*, was represented by the product of two Gaussian likelihoods, representing the probability of the neural data evoked by the first stimulus, given the starting position of the candidate trajectory, and the probability of the neural data evoked by the second stimulus, given the endpoint of the candidate trajectory. Each likelihood was centered at the actual location of the corresponding stimulus:
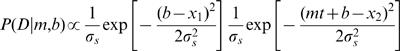
8where *x_1_* and *x_2_* represent the actual first and second stimulus positions, respectively; *t* represents IST; and the standard deviation *σ_s_* is the same for each likelihood. Actual ISD, *l*, was *x_2_* -*x_1_*, and actual velocity, *v*, was *l/t*.

Substituting Equations 7 and 8 into Equation 6 provided an expression for the posterior probability of each candidate trajectory:
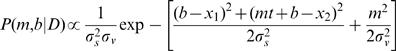
9


The intensity plots of Fig. 2B, left column, were obtained by computing the values of P(m,b), P(D|m,b), and P(m,b|D) from Equations 7, 8, and 9, respectively, for a range of *m* and *b* values, using *σ_s_* = 1 cm, and *σ_v_* = 10 cm/s. The plots in Fig. 2B, right column, were derived numerically from those shown in the left column.

The mode of the posterior was found analytically by setting to zero the partial derivatives of the exponent of Equation 9 with respect to *m* and *b*. This resulted in expressions for perceived velocity, *v'* (the value of *m* at the mode of the posterior; Equation 3) and perceived ISD, *l'* (i.e., *v'* *t*; Equation 1). The partial derivative of Equation 3 with respect to *t* was set to zero to derive Equations 4 and 5.

### Basic Model with Spatial Attention (Fig. 5)

The basic model was extended to allow *σ_s_* to take on different values at the two stimulus positions. The prior (Equation 7) was the same as that for the basic model, but the likelihood included independent spatial uncertainty terms, *σ_s1_* and *σ_s2_*, representing the standard deviations of the Gaussian likelihoods evoked by the first and second stimuli, respectively. This modification resulted in the posterior:
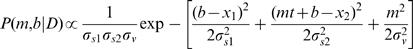
10


The mode of the posterior was found by setting to zero the partial derivatives of the exponent of Equation 10 with respect to *m* and *b*. This resulted in expressions for perceived velocity, *v'* (the value of *m* at the mode of the posterior) and perceived ISD, *l'* (i.e., *v't*):
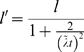
11where the modified *λ* replaces *σ_s_* with the root-mean-square of *σ_s1_* and *σ_s2_*:
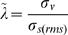
When the spatial uncertainties are equal, Equation 11 reduces to Equation 1.

The value of *b* at the mode of the posterior (the perceived position of the first stimulus), together with *l'*, was used to calculate the midpoint of the perceived trajectory. The midpoint of the perceived trajectory was found to be displaced from that of the real trajectory, (*x_1_*+*x_2_* )/2, by a distance *Δl* given by:
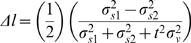
12


Equation 12 shows that as the difference between *σ_s1_* and *σ_s2_* increases, the perceived midpoint more closely approaches the position of the preferentially attended (smaller *σ_s_* ) location. When *σ_s1_* equals *σ_s2_*, the extended basic model reduces to the original basic model, and *Δl* = 0, indicating that the perceived and real trajectories share the same midpoint.

### Full Model (Fig. 6)

The full model admits temporal as well as spatial uncertainty. Each candidate trajectory was described by a velocity, *m*; a first stimulus position, *b*; a starting stimulus time, *t_1_*; and a duration, *τ*. As in the basic model, each Gaussian spatial likelihood was centered at the actual location of the corresponding stimulus. In addition, analogous temporal likelihoods were centered at the actual times of the corresponding stimuli (The actual time of the first stimulus was defined as zero, and that of the second stimulus, as *t*).

The trajectory likelihood was then:
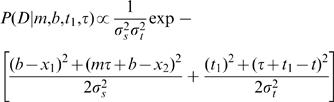
13


As in the basic model, the prior reflected an expectation for slow movement:
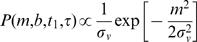
14Note that Equation 14 has the same form as Equation 7, reflecting the use of uniform priors for all parameters except velocity.

The posterior, proportional to the product of prior and likelihood, was:
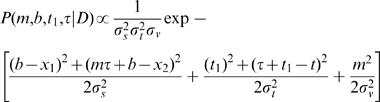
15


The mode of the posterior was found by setting to zero the partial derivatives of the exponent of Equation 15 with respect to *m, b, t_1_*, and *τ*. This resulted in expressions for perceived IST, *t'* (the value of *τ* at the mode of the posterior); perceived velocity, *v'* (the value of m at the mode of the posterior); and perceived ISD, *l'* (i.e., *v't'* ; Equation 2):

The equation relating t to t' was found to be:
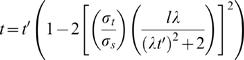
16


Equation 16 was solved numerically for *t'*, given values for *t, l, λ, σ_t_* and *σ_s_*. The equation shows that real IST, *t*, is less than perceived IST, *t'*; that is, the model experiences perceptual time dilation. Note that *t'* tends towards *t* in the limit of large *σ_s_*; that is, relatively little time dilation occurs on areas of skin with poor spatial acuity. Finally, Equation 16 yields *t* = *t'* when *σ_t_* is set to zero, as the full model then reduces to the basic model, which perceives time veridically.

### Data Extraction

The data plotted in Figures 3B and C were taken from Table 1 of reference [17] and Table 3 of reference [18], respectively. The data plotted in Figures 3A, D, and E were extracted from Figure 1 of reference [12], Figure 1 of reference [19], and Figure 6 of reference [20], respectively, using GraphClick v. 12.9 (Arizona Software).
